# QSTR Models in Dioxins and Dioxin-like Compounds Provide Insights into Gene Expression Dysregulation

**DOI:** 10.3390/toxics12080597

**Published:** 2024-08-17

**Authors:** Elisa G. Eleazar, Andrei Raphael M. Carrera, Janus Isaiah R. Quiambao, Alvin R. Caparanga, Lemmuel L. Tayo

**Affiliations:** 1 School of Graduate Studies, Mapua University, Manila 1002, Philippines; egeleazar@mapua.edu.ph (E.G.E.); armcarrera@mapua.edu.ph (A.R.M.C.); arcaparanga@mapua.edu.ph (A.R.C.); 2 School of Chemical, Biological, and Materials Engineering and Sciences, Mapua University, Manila 1002, Philippines; jiquiambao2@gmail.com; 3 Department of Biology, School of Health Sciences, Mapua University, Makati 1200, Philippines

**Keywords:** dioxins, furans, QSTR, molecular docking, molecular dynamics

## Abstract

Polychlorinated dibenzo-p-dioxins and polychlorinated dibenzo-p-furans (PCDD/Fs) are a group of organic chemicals containing three-ring structures that can be substituted with one to eight chlorine atoms, leading to 75 dioxin and 135 furan congeners. As endocrine-disrupting chemicals (EDCs), they can alter physiological processes causing a number of disorders. In this study, quantitative structure–toxicity relationship (QSTR) studies were used to determine the correlations between the PCDD/Fs’ molecular structures and various toxicity endpoints. Strong QSTR models, with the coefficients of determination (r^2^) values greater than 0.95 and ANOVA *p*-values less than 0.0001 were established between molecular descriptors and the endpoints of bioconcentration, fathead minnow LC50, and *Daphnia magna* LC50. The ability of PCDD/Fs to bind to several nuclear receptors was investigated via molecular docking studies. The results show comparable, and in some instances better, binding affinities of PCDD/Fs toward the receptors relative to their natural agonistic and antagonistic ligands, signifying possible interference with the receptors’ natural biological activities. These studies were accompanied by the molecular dynamics simulations of the top-binding PCDD/Fs to show changes in the receptor–ligand complexes during binding and provide insights into these compounds’ ability to interfere with transcription and thereby modify gene expression. This introspection of PCDD/Fs at the molecular level provides a deeper understanding of these compounds’ toxicity and opens avenues for future studies.

## 1. Introduction

**Figure 1:**
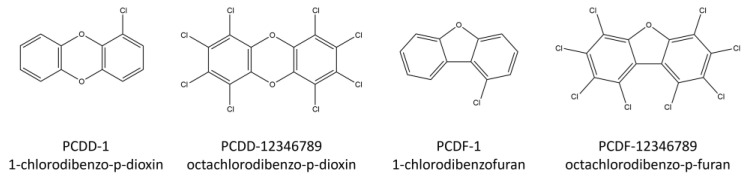
Structures of some PCDD/F compounds.

Polychlorinated dibenzo-p-dioxins and polychlorinated dibenzo-p-furans (PCDD/Fs) are a group of organic chemicals containing three-ring structures that can be substituted with one to eight chlorine atoms, leading to 75 dioxin and 135 furan congeners. Figure 1 shows the structure of the simplest and most complex PCDD/Fs.

PCCD/Fs can be released from natural processes, like volcanic eruptions and forest fires, but their presence in the environment is mainly due to atmospheric emissions from various industrial processes, including waste incineration, combustion-related activities, chlorine bleaching of paper pulp, and metal smelting [1,2]. Human exposure occurs via food or the environment and results in the uptake of a large number of these compounds, leading to retention in human blood, tissues, and milk [3,4,5]. These compounds have been listed as two of the initial persistent organic pollutants (POPs) identified during the Stockholm Convention. As such, they remain intact for exceptionally long periods of time, become widely distributed throughout the environment, are able to bioaccumulate and bioconcentrate, and are toxic to both humans and wildlife [6]. Toxicological studies have been carried out on PCDD/Fs using rats, mice, hamsters, and other organisms [6,7,8,9]. However, such studies do not offer insight into the underlying molecular interactions causing their toxicity. This brings to light the importance of quantitative structure–toxicity relationship (QSTR) studies.

QSTR studies involve the development of predictive models of biological activities as a function of the structural and molecular information of compounds. This technique recognizes the fact that pollutants with similar chemical structures are likely to have similar physicochemical properties and thereby exhibit equivalent toxicological behavior. Several studies have been performed on the relationship between molecular structure and various toxicity endpoints. The molecular structures of aromatic hydrocarbons and organophosphates have been correlated to toxicity in rodents [10,11,12,13]. QSTR models showing the relationship between agrochemicals, pesticides, and polychlorinated naphthalenes and toxicity to *Daphnia magna* have been reported [14,15,16,17]. Studies have likewise been performed on the association between polycyclic aromatic hydrocarbons and the molecular fragments of textile dyes, personal care products, and plant protection products to algae, fish, and honeybees [18,19,20,21].

**Figure 2:**
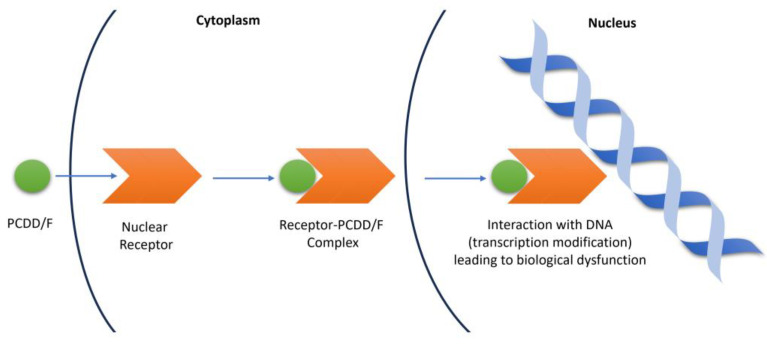
Mechanism of gene expression interference by PCDD/Fs.

The toxic nature of PCDD/Fs makes it important for QSTR studies to be conducted, especially in aquatic organisms. These organisms normally find their way into the food chain, leading to increased human exposure via bioconcentration and bioaccumulation. Furthermore, PCDD/Fs have been identified as endocrine-disrupting chemicals (EDCs). EDCs are able to interfere with the synthesis, secretion, transport, metabolism, binding action, or elimination of natural-borne hormones that are responsible for homeostasis, reproduction, and developmental processes, by exerting action through nuclear receptors [22,23]. Nuclear receptors mediate the transcriptional activities of genes. These are intracellular proteins that bind to small molecules or ligands and, upon activation, translocate to the cell nucleus where they modulate gene expression through interactions with DNA. As EDCs, PCDD/Fs can block and alter the physiological processes and pathways of the nuclear receptors by displacing their natural ligands and inducing conformational changes. The receptor–PCDD/F complex can then interact with DNA, resulting in modifications in gene expression. Gene expression variations modify cell behavior and alter the normal functions of tissues and organs [24]. Figure 2 shows the mechanism of the gene expression interference by PCDD/Fs.

This study looks into the interaction of PCDD/Fs on eight nuclear receptors: progesterone, estrogen, vitamin D, androgen, thyroid-α, thyroid-β, retinoic acid-α, and retinoic acid-β. The progesterone receptor (PR) regulates female development and reproduction. Its functions are important in normal breast development and menstrual cycle regulation and are implicated in diseases such as endometriosis and infertility [25,26,27]. In conjunction with PR, the estrogen receptor (ER) plays a key role in the menstrual cycle and pregnancy support. Abnormal ER signaling is associated with breast and ovarian cancers [28,29,30]. The vitamin D receptor (VDR), upon creating a complex with vitamin D, regulates immune response and maintains calcium and phosphorus homeostasis necessary for bone health. Autoimmune diseases, cancer, and cardiovascular disorders are associated with irregular functions of the VDR [31,32,33,34,35]. The androgen receptor (AR) is responsible for the secretion of androgen hormones and maintaining male characteristics, including muscle development and voice deepening. The disrupted signaling of the AR is linked to androgen insensitivity syndrome and prostate cancer [36,37,38]. The thyroid receptor (THR) is known to be important in development regulation and lipid metabolism. Defects in THR functions have been reported to be involved in hypo- and hyperthyroidism, as well as thyroid hormone resistance [39,40,41]. The retinoic acid receptor (RAR) regulates embryonic, skin, and vision development. The aberrant activation and dysfunction of RAR lead to congenital malformations and skin diseases [42,43,44]. Table 1 shows the physiological functions of these nuclear receptors, along with the effects of the EDCs’ interference. The impacts of biological dysfunction emphasize the importance of studying how PCDD/Fs interact with these nuclear receptors.

This study focuses on the PCDD/Fs’ molecular and structural identities, leading to correlations to reference toxicity endpoints. Molecular docking studies were performed to predict the possible molecular interactions between the nuclear receptors and the PCDD/F ligands that may lead to regulatory networks in the biological processes. Molecular dynamics simulations were then conducted to understand the changes occurring at the cellular level and verify the stability of the receptor–ligand complexes. This introspection at the molecular level allows for a better understanding of these compounds’ toxicity and addresses the need for a thorough systematic study of PCDD/Fs.

## 2. Materials and Methods

**Figure 3:**
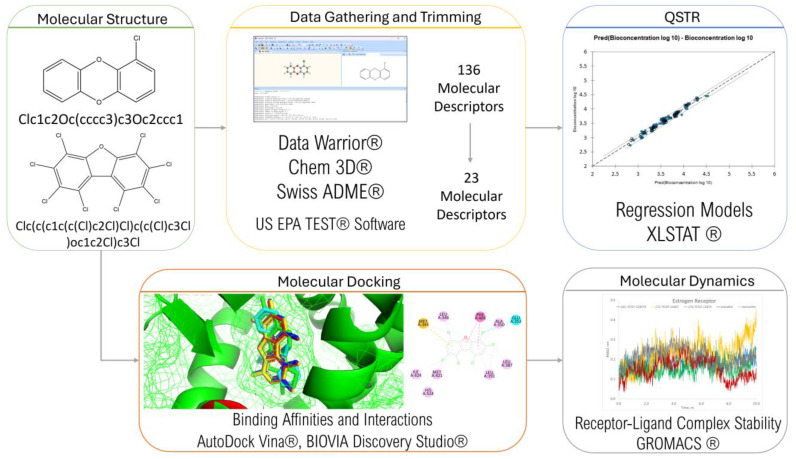
Research framework.

The framework of this study is summarized in Figure 3. Data were gathered and trimmed, the results of which were used in QSTR modeling. Molecular docking was then conducted to determine the PCDD/Fs’ ability to bind with different nuclear receptors. The top-binding PCDD/Fs were then subjected to further analysis using molecular dynamics.

### 2.1. Data Gathering and Trimming

Data Warrior^®^ [45], a universal data analysis and visualization program, was used to generate the 3D structures and Simplified Molecular Input Line Entry System (SMILES) of the 210 PCDD/Fs. Several online databases were used to determine the properties of the congeners: Data Warrior^®^ for the constitutional descriptors, Chem3D^®^ 20.1 (Revvity Signals Software) for the molecular energies, and SwissADME^®^ [46] for the assessment descriptors. Molecular activities were determined using the Toxicity Estimation Software Tool (TEST^®^) version 5.1 from the US EPA [47] and were then used as toxicity endpoints. To minimize redundancy, variables with constant values were omitted, and a correlation matrix was created on the remaining variables to omit those with high correlation.

### 2.2. Regression and Principal Component Analysis

XLSTAT^®^ [48] was used to perform regression analysis and principal component analysis. Multiple linear regression (MLR) was carried out to explore the linear relationship between the molecular descriptors and each toxicity endpoint. A training set of 150 PCDD/Fs and a test set of 60 randomly selected PCDD/Fs were used.

The contributory descriptors identified during regression were subjected to principal component analysis (PCA) for further exploration of their correlation and contribution, providing insight into the underlying correlations.

### 2.3. Molecular Docking Studies

Molecular docking simulations were performed to examine the intermolecular interactions between the PCDD/Fs and several target receptors, including progesterone (PR), estrogen (ER), vitamin D (VDR), androgen (AR), thyroid (THR), and retinoic acid (RAR), with PDB accession codes 1A28 [49], 1A52 [50], 1DB1 [51], 1E3G [52], 1NAV [53], 1NAX [53], 1DKF [54], and 1XDK [55], respectively. AutoDock Tools^®^ [56] was used to preprocess the receptor files. Water, heteroatoms, and suspended ligands were deleted, polar hydrogen was added to improve the performance of the cavity method, and AutoGrid^®^ was used to determine the grid box dimensions. Molecular docking was carried out using AutoDock Vina^®^ [57,58], and visualization was carried out using AutoDock Tools^®^, PyMol^®^ [59], and BIOVIA Discovery Studio^®^ [60].

### 2.4. Molecular Dynamics Simulation

The top-binding PCDD/Fs were subjected to molecular dynamics simulations to examine the stability of the receptor–ligand complexes. The simulations were carried out using GROMACS [61] for 100 ns. The docked receptor–ligand complexes were used as input files. The simulations were conducted using the CHARMM36 all-atom force field and the CHARMM-modified transferable intermolecular interaction potential 3 points (TIP3P) [62,63]. The models were neutralized via the addition of water molecules and counter ions as a solvent to reflect physiological conditions.

## 3. Results and Discussion

### 3.1. Data Gathering and Trimming

One hundred twenty-six molecular descriptors were collected using Data Warrior^®^, Chem3D^®^, and SwissADME^®^. The uniform and redundant variables were eliminated, resulting in 23 descriptors, 14 of which were constitutional descriptors, 8 were energy descriptors, and 1 was an assessment descriptor. The constitutional descriptors included molecular weight (MW), number of chlorine atoms (Cl), number of symmetric atoms (NSA), total surface area (TSA), globularity SVD (GSVD), globularity volume (GV), van der Waals surface area (VDWSA), van der Waals volume (VDWV), shape index (SI), molecular flexibility (MF), molecular complexity (MC), log P, log S, and molar refractivity (MR). The energy descriptors included stretch energies (SE), bend energies (BE), torsion energies (TE), non-1,4-van der Waals energies (NVDW), 1,4-van der Waals energies (VDWE), dipole–dipole energies (DDE), and total energy (TTE). The assessment descriptor was synthetic accessibility (SA). The molecular descriptors can be found in Table S1 of the Supplementary Materials.

The US EPA Toxicity Estimation Software Tool^®^ (TEST) was used to calculate the molecular activities of PCDD/Fs on several endpoints, four of which were organism-specific toxicities. These included (1) toxicity to *Tetrahymena pyriformis*, a free-living unicellular eukaryote from the phylum Ciliophora, in terms of 50% growth inhibition after a 48 h exposure (IGC50); (2) toxicity to *Daphnia magna*. a planktonic crustacean from the phylum Arthropoda, in terms of 50% lethality after a 48 h exposure (LC50); (3) toxicity to *Pimephales promelas*—fathead minnow fish from the phylum Chordata, in terms of 50% lethality after a 96 h exposure (LC50); and (4) toxicity to rat, in terms of 50% lethality (LD50) [64]. The other endpoints included bioconcentration, developmental toxicity, and Ames mutagenicity.

### 3.2. QSTR Models

Quantitative structure–toxicity relationship (QSTR) involves a modeling approach similar to quantitative structure–activity relationship (QSAR) but with a focus on adverse molecular activities. In this study, QSTR was used to examine the relationship between various molecular descriptors and toxicity endpoints. Three models were developed using the multiple linear regression (MLR) technique. These were models for bioconcentration, fathead minnow LC50, and *Daphnia magna* LC50:

(1) $${BC}_{log10}=5.986+0.519\left(Cl\right)+0.761\left(GSVD\right)+0.016\left(VDWS\right)-0.031\left(VDWV\right)-1.086\left(SI\right)+0.102\left(SB\right)-0.221\left(SA\right)$$

(2) $${FM}_{-\log10}=2.499+1.318(MC)+0.543(logP)+0.192(SB)$$

(3) $${DM}_{-\log10}=16.189-0.040\left(MW\right)+1.687\left(Cl\right)-6.764\left(GV\right)+2.416\left(MC\right)+0.012\left(NVDW\right)-0.012\left(TE\right)$$

**Figure 4:**
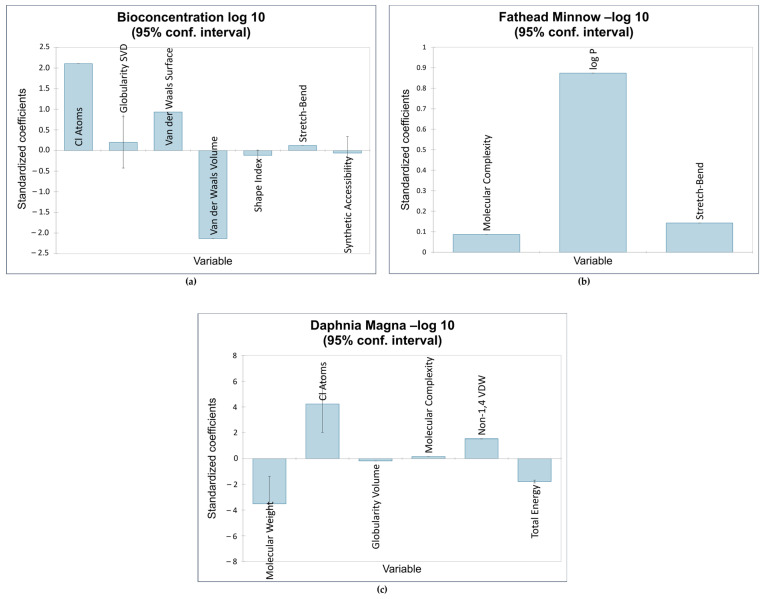
Impact of molecular descriptors on toxicity endpoints: (**a**) bioconcentration; (**b**) fathead minnow LC50; (**c**) *Daphnia magna* LC50.

The first QSTR model provided the correlations between the descriptors Cl, GSVD, VDWS, VDWV, SI, SB, and SA and the bioconcentration endpoint. Of the seven descriptors, the number of chlorine atoms was the most influential. This is consistent with the results obtained by Bordajandi et.al. [65], who revealed that samples with a high degree of chlorination were predominant in sea fish. This also agrees with the model presented by Bertato et.al. [66], who indicated that the presence of chlorine atoms increases bioconcentration. Furthermore, the more chlorine atoms are attached to dioxins and dioxin-like compounds, the higher the lipophilicity and melting point, and the lower the solubility in water. This makes these compounds vastly soluble in fat, leading to bioaccumulation and bioconcentration [67]. This model is also highly influenced by van der Waals volume, which is the average molecular volume when considering van der Waals forces. The molecular volume contributes to the compounds’ hydrophobicity [20], validating the negative impact of van der Waals volume on bioconcentration. The next model showed a positive relationship between fathead minnow LC50 and the following descriptors: MC, log P, and SB. The most influential descriptor was logP, a measure of a compound’s hydrophobicity. This is consistent with QSTR models reported in the literature relating the hydrophobicity of various organic compounds to acute toxicity in fish [16,68,69,70,71,72]. Figure 4 shows the impact of the standardized coefficients of the molecular descriptors on the three toxicity endpoints.

**Figure 5:**
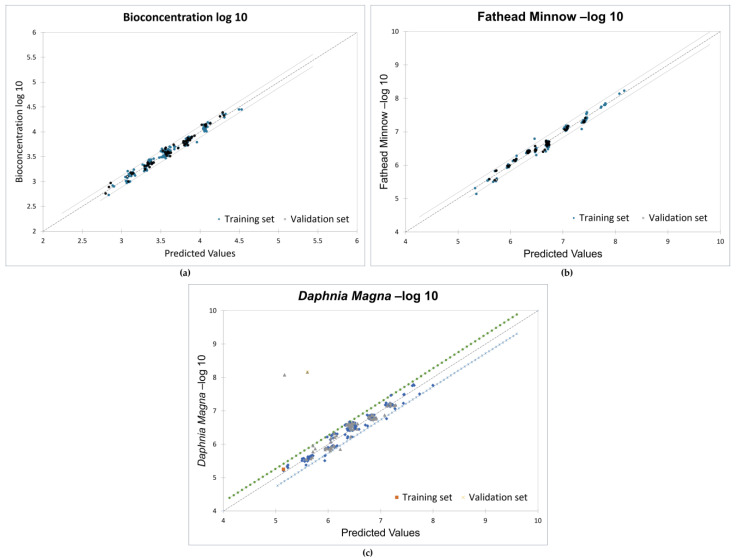
Validation plots for MLR models: (**a**) bioconcentration; (**b**) fathead minnow LC50; (**c**) *Daphnia magna* LC50.

The models presented in this study were developed using constitutional, energy, and assessment descriptors as these descriptors have been linked to molecular reactivity, adsorptivity, and toxicity [73]. All the 210 PCDD/F congeners (divided into 70% training set and 30% test set) were used to obtain strong correlations between the descriptors and the toxicity endpoints. Favorable goodness-of-fit scores with coefficients of determination (r^2^) greater than 0.95 and ANOVA *p*-values less than 0.0001 were recorded. Figure 5 shows the validation plots for the models.

The results of the significance test indicate the significant impact of the descriptors at the 95% confidence interval, as shown in Table 2. The slopes of the descriptors have probabilities less than 5%, and the adjusted correlation coefficients are 0.975, 0.979, and 0.95 for the three endpoints, respectively. The root-mean-square error (RMSE) values for all training and validation sets are below 0.5. This confirms the strong relationship between the toxicity endpoints and the molecular descriptors.

### 3.3. Principal Component Analysis

**Figure 6:**
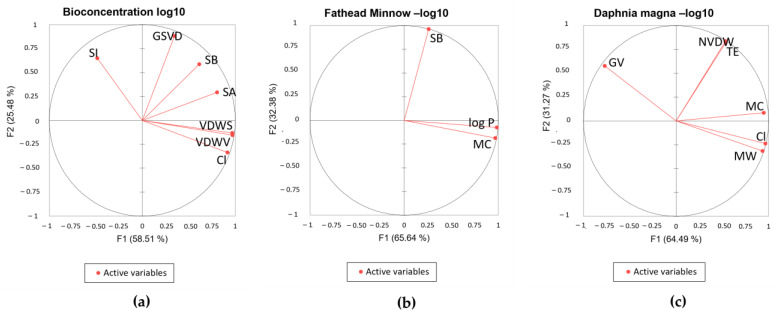
Biplots of QSTR models: (**a**) bioconcentration log 10; (**b**) fathead minnow LC50 log 10; (**c**) *D. magna* LC 50 log 10.

Principal component analysis (PCA) was conducted to examine the correlation between the significant descriptors in the QSTR models. As a multivariate analysis, PCA is a powerful dimensional reduction technique that shows advantages in data simplification [74]. The dimensionality of the variables was reduced and visualized in the PCA biplots, as shown in Figure 6. Figure 6a shows a high correlation between the number of chlorine atoms and the van der Waals surface area and volume. Less correlation can be seen between GSVD, SB, and SA. Figure 6b shows a high correlation between MC and log P. Figure 6c shows a high correlation between MW, Cl, and MC.

### 3.4. Molecular Docking Studies

**Figure 7:**
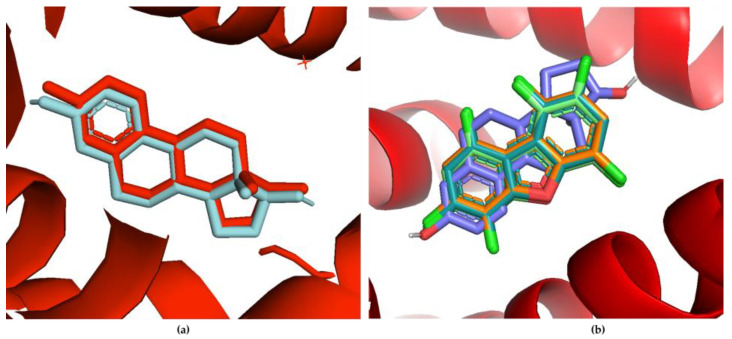
(**a**) Redocked estradiol ligand (cyan) compared with the co-crystallized ER with the same ligand (red); (**b**) overlay of the binding poses of the top-binding PCDD/Fs and estradiol on the same estrogen-binding site.

Molecular docking studies were performed via AutoDock Vina^®^ to examine the molecular interactions between the PCDD/Fs and several nuclear receptors. The docking protocol was validated by redocking the estradiol ligand and superimposing it with the co-crystallized ER [75]. The redocked ligand shows a similar pose on the same active site, denoting homology. Figure 7a shows the redocking of estradiol onto the estrogen-binding site. The docking poses of the top-binding PCDD/Fs and the natural ligand estradiol onto ER on the same docking site are shown in the overlay figure in Figure 7b.

**Figure 8:**
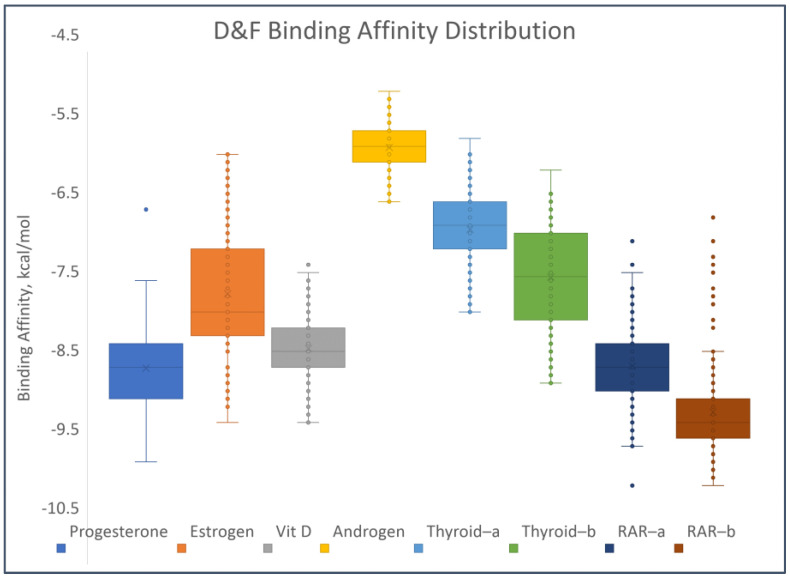
Distribution of binding affinities of the PCDD/Fs to selected nuclear receptors.

Figure 8 shows the distribution of the binding affinities of the PCDD/Fs to the different receptors. The boxes in the figure encompass the calculated binding affinities from the 25th to the 75th percentile. This means that 50% of the calculated binding affinities for each receptor fall within the values bound by each box. Therefore, there is a larger range of binding affinities to ER and THR-α receptors relative to the other receptors. Similarly, the range of affinities to the androgen receptor is the smallest. Outlier values are present in the binding affinities to PR, VDR, and RAR. The lowest median values of the PCDD/F binding affinities were observed for RAR, PR, VDR, and ER. This implies ease of binding of the PCDD/Fs to these receptors, relative to THR and AR. To substantiate this observation, the same docking methods were performed between the nuclear receptors and their natural agonistic and antagonistic ligands for reference and comparison. The results show comparable, and in some cases better, binding affinities of the PCDD/Fs relative to the reference ligands. For PR, 139 out of the 210 PCDD/Fs have better binding affinities than its natural ligand mifepristone. For THR, 84% of the PCDD/Fs have better binding affinities than the reference ligand NH-3. In the case of RAR, all 210 PCDD/Fs have affinities stronger than or the same as that of the natural ligand retinoin. The complete results of the molecular docking studies can be found in Table S2 of the Supplementary Materials.

**Figure 9:**
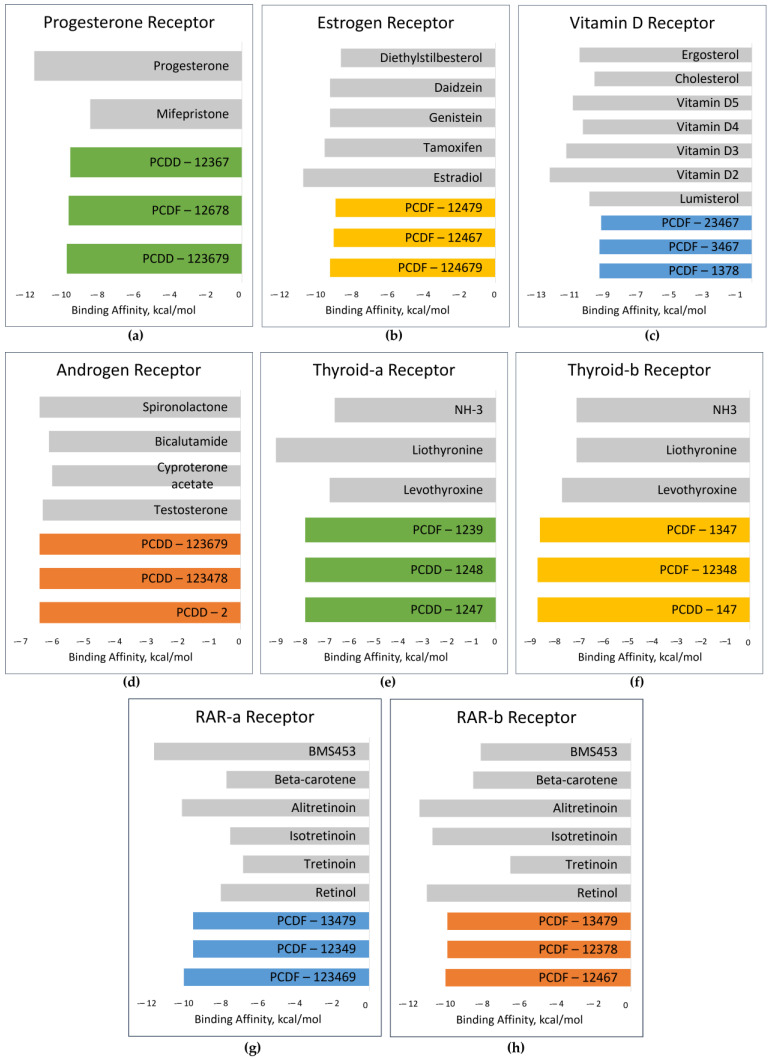
Binding affinities of top-binding PCDD/Fs (colored) in comparison with those of the natural ligands (gray): (**a**) PR; (**b**) ER; (**c**) VDR; (**d**) AR; (**e**) THR-α; (**f**) THR-β (**g**) RAR-α; (**h**) RAR-β.

For PR, the top-binding PCDD/F is PCDD-123679, with a binding affinity of −9.7 kcal/mol. The binding affinities of its natural ligands, namely progesterone and mifepristone, are −11.5 and −8.4 kcal/mol, respectively. This could mean that PCDD-123679 binds better to PR than mifepristone. Figure 9 shows the binding affinities of the top-binding PCDD/Fs in comparison with the binding affinities of some natural ligands of the receptors. All three top-binding PCDD/Fs to AR have binding affinities better than all the natural ligands tested. This is the same for THR-β. All top-binding PCDDF/s to all the receptors have binding affinities better than −6 kcal/mol. When the binding affinity energy value is less than −6 kcal/mol, binding is more likely to appear [76]. At this point, it can be surmised that PCDD/Fs possess the ability to bind with nuclear receptors. They can potentially displace or compete with the natural ligands and thereby trigger action.

**Figure 10:**
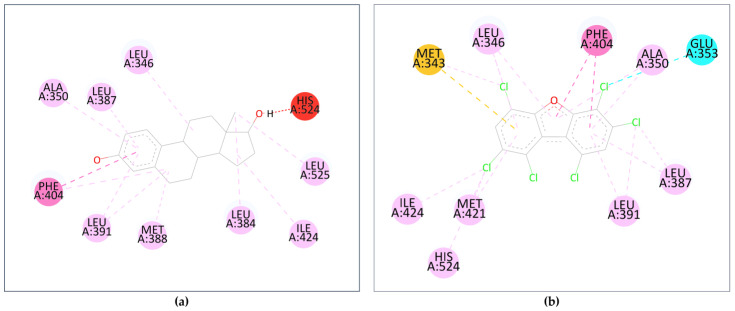
Interactions between the amino acid residues and (**a**) estradiol; (**b**) PCDF-124679. The dashed lines represent interactions between the ligand and the receptor’s amino acid residues (colored orbs). The orb colors represent the different types of interactions: red—unfavorable donor-donor interaction; dark pink—pi–pi T-shaped; yellow—pi–sulfur; cyan—halogen; soft pink—alkyl and pi–alkyl.

Further analysis of the top-binding PCDD/Fs was carried out by probing the interactions between the ligands and the receptor amino acid residues, using the natural ligands as reference. Visualization was carried out using BIOVIA Discovery Studio^®^, samples of which are shown in Figure 10. Figure 10a shows the amino acid residue interactions in estradiol, a natural ER ligand, and Figure 10b shows the interactions in the top-binding PCDD/F, PCDF-124679, to the same receptor. The binding affinities are attributed, among others, to the presence of hydrophobic interactions between the ligands and the receptors. Hydrophobic interactions occur between alkyl groups of both ligands and receptors. Other interactions occur as pi–alkyl interactions or pi–pi T-shaped interactions (alkyl–pi hydrogen interactions). Hydrophobic interactions can increase the binding affinity between receptor–ligand interfaces [77].

Several common intermolecular interactions were observed between the top-binding PCDD/Fs and ER (6 out of 9), as shown in Figure 10b. These include three alkyl bonds between PCDF-124679 and the estrogen receptor’s Leu346, Leu391, and Ile424 amino acids, as well as three pi–alkyl bonds between PCDF-124679 and ER’s Ala350, Leu387, and Phe404 amino acids. The pi–sulfur bond between PCDF-124679 and ER’s Met343 amino acid allows for greater flexibility and induced fit, rationalizing the PCDD/F’s binding affinity.

**Figure 11:**
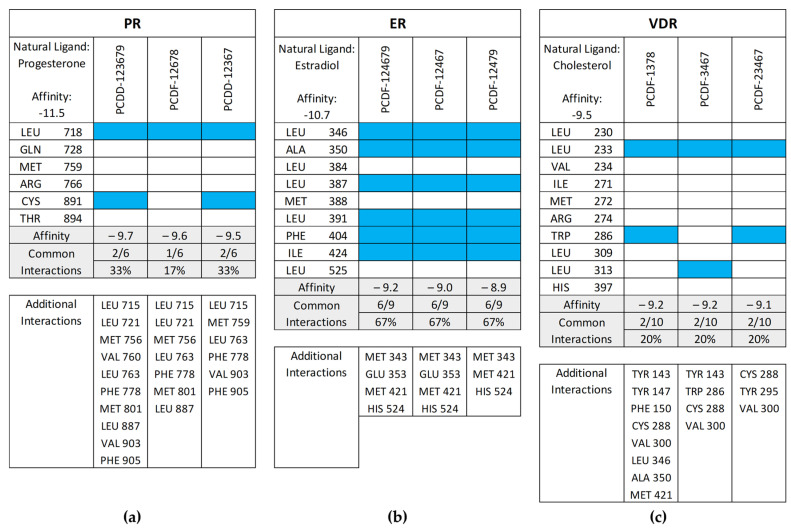

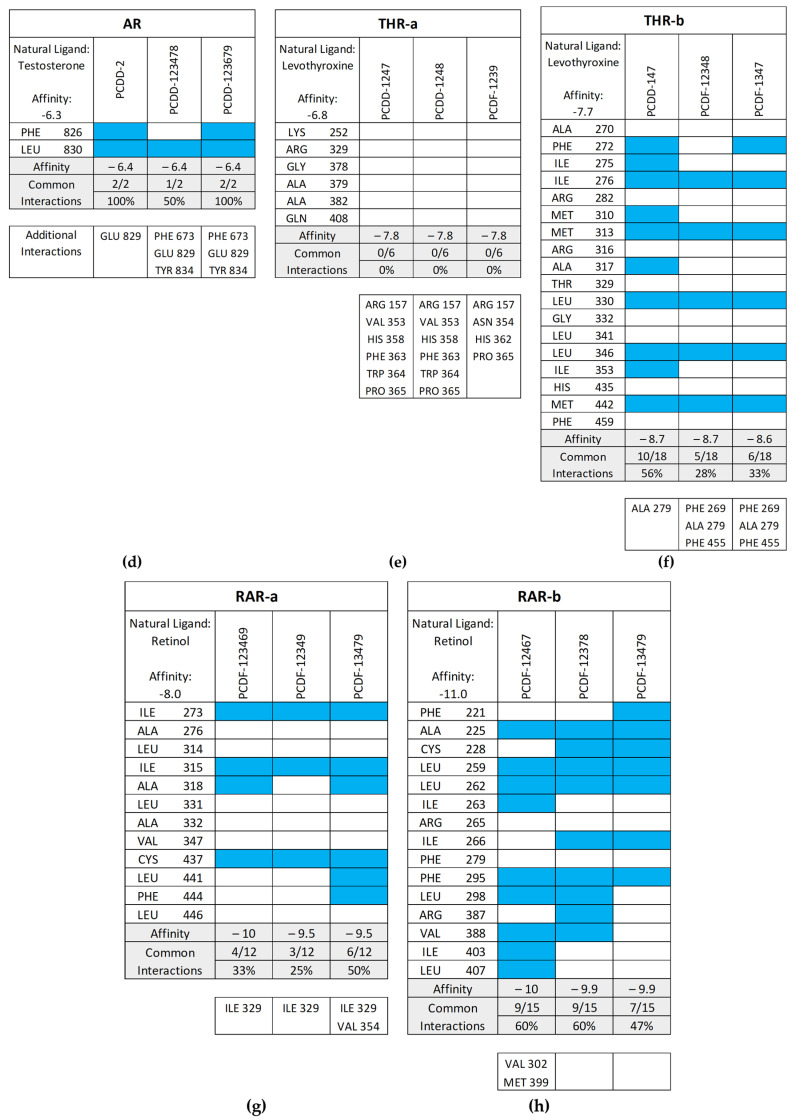
Common interactions (represented by the blue shade) between the top-binding PCDD/Fs and the receptors’ amino acid residues relative to the receptors’ natural ligands: (**a**) PR; (**b**) ER; (**c**) VDR; (**d**) AR; (**e**) THR-α; (**f**) THR-β; (**g**) RAR-α; (**h**) RAR-β. The amino acid residues listed at the bottom of the commonality plots refer to the additional hydrophobic interactions between the PCDD/Fs and the nuclear receptors.

Figure 11 shows the common interactions between the top-binding PCDD/Fs and the receptors’ amino acid residues relative to the reference ligands, as well as the additional hydrophobic interactions observed between the PCDD/Fs and the nuclear receptors. In the case of PR, two out of the six interactions were observed to be common between the top-binding PCDD/F, PCDD-123679, and the receptor. While this may have affected the PCDD/F’s binding affinity with the receptor, several other hydrophobic interactions were observed, including those to the Leu715, Leu721, Met756, Val760, Le763, Phe778, Met801, Leu887, Val903, and Phe905 amino acid residues. These are known to be hydrophobic and improve binding between the receptor and the ligand.

### 3.5. Molecular Dynamics Simulations

The top-binding PCDD/Fs were subjected to molecular dynamics simulations to examine the changes occurring at the molecular level in the protein–ligand system and gain insight into the stability of the complexes. This study used the root-mean-square deviation (RMSD), root-mean-square fluctuation (RMSF), and interaction energies as parameters for analyzing the stability of the protein–ligand complexes.

**Figure 12:**
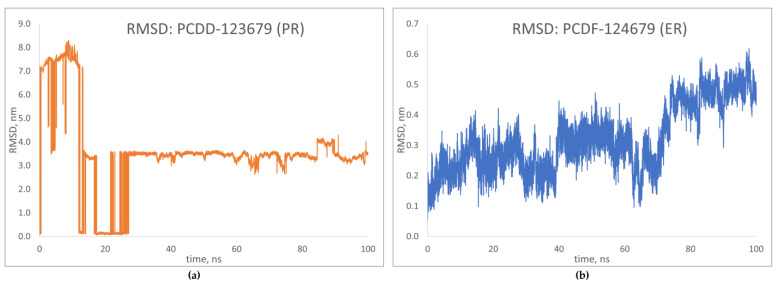
RMSD plots of top-binding PCDD/Fs: (**a**) PCDF-123679 to PR; (**b**) PCDF-124679 to ER.

RMSD predicts the changes in conformation occurring in the protein backbone during simulation [24]. The results for the top-binding PCDD/Fs, PCDD-123679 bound to the progesterone receptor, and PCDF-124679 bound to the estrogen receptor are shown in Figure 12. Large deviations were observed for PCDD-123679 (PR), although the complex was found to have achieved equilibrium around 30 ns. The results for PCDFF-124679 (ER) show deviations that are relatively smaller, but an increasing trend is observed around 75 ns of simulation, showing increased deviation. These results show conformational changes in the receptor upon binding with the PCDD/Fs. The results of the molecular dynamics simulations can be found in Table S3 of the Supplementary Materials.

**Figure 13:**
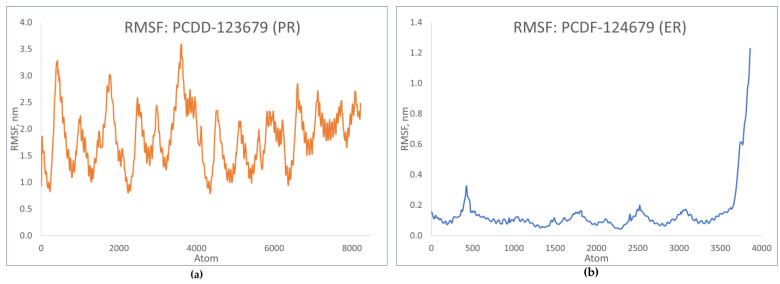
RMSF plots of top-binding PCDD/Fs: (**a**) PCDF-123679 to PR; (**b**) PCDF-124679 to ER.

RMSF shows how the coordinates of the Cα atoms fluctuate relative to their average position during simulation [23]. Figure 13 shows the fluctuation patterns of the top-binding PCDD/Fs with the progesterone and estrogen receptors. The RMSF values for PCDD-123679 indicate significant fluctuations during simulation, showing dynamic behavior during binding. In the case of PCDF-124679, significant flexibility is observed in the terminal atoms. These fluctuation patterns are similar to those of the receptor–natural ligand complexes, indicating a similar response to that of natural ligand binding, which in turn may correspond to the similar activation of signaling events.

**Figure 14:**
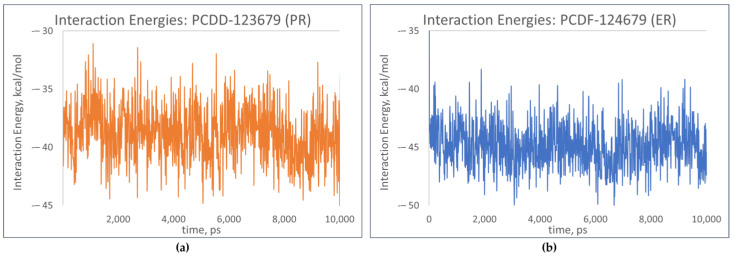
Total interaction energies of top-binding PCDD/Fs: (**a**) PCDF-123679 to PR; (**b**) PCDF-124679 to ER.

Further analysis of the receptor–ligand complexes was performed by examining the simulation energy using the short-range Coulomb (Coul-SR) energy and the short-range Lennard–Jones energy (LJ-SR). These short-range energies take into consideration the interaction energies between atoms or residues based on their distance of separation and are dependent on how much of the receptor is “in contact” with the ligand [24]. Figure 14 shows slight fluctuations in the interaction energies of the top-binding PCDD/Fs, signifying the PCDD/Fs’ capability to form strong, thermodynamically stable complexes with the nuclear receptors.

The molecular dynamics simulation results provide insights into the dynamic properties of the receptor–PCDD/F complexes. The equilibrium shifts may correspond to transitions, which in turn can lead to a cascade of biological activities. Similarities between the flexibility of the receptor–natural ligand complexes and that of the receptor–PCDD/F complexes suggest similar responses in terms of DNA interaction, leading to gene expression dysregulation.

## 4. Conclusions

A systematic study on the molecular properties related to the toxicity of polychlorinated dioxins and furans was carried out. Strong correlations, with coefficients of determination greater than 0.95 and ANOVA *p*-values less than 0.0001, were established between several toxicity endpoints (bioconcentration, LC50 toward fathead minnow, and LC50 toward *Daphnia magna*) and the molecular and structural information of PCDD/Fs. The number of chlorine atoms and van der Waals volume exerted the most influence on the bioconcentration model. Moreover, log P yielded the most significant information on the variability of the fathead minnow LC50, while molecular weight and the number of chlorine atoms revealed the most significant information on the variability of *Daphnia magna* LC50.

The molecular docking studies demonstrate the ability of the PCDD/Fs to bind to several nuclear receptors, including the progesterone, estrogen, vitamin D, androgen, thyroid-α, thyroid-β, retinoic acid-α, and retinoic-β receptors. The results show comparable, and in some instances better, binding affinities of the PCDD/Fs toward the receptors relative to their natural agonistic and antagonistic ligands, signifying possible interference with the receptors’ natural biological activities. The interaction analysis on the docked receptor–ligand complexes highlights the existence of some hydrogen bonds and hydrophobic interactions, elucidating the occurrence of strong binding affinities between the PCDD/Fs and the nuclear receptors.

The molecular dynamics simulations show the changes occurring at the molecular level in the receptor–ligand complexes. The thermodynamically stable receptor–PCDD/F complexes were found to respond similarly to those of the receptor–natural ligand complexes, signifying the ability of PCDD/Fs to interfere with transcription and thereby modify gene expression. This introspection of PCDD/Fs at the molecular level provides a deeper understanding of these compounds’ toxicity and opens avenues for future studies.

## Figures and Tables

**Table 1:** Summary of physiological functions and dysfunction impacts of various nuclear receptors.

Receptor	Physiological Function	Dysfunction Impacts
PR	Breast development	Endometriosis and infertility
	Menstrual cycle regulation	
ER	Menstrual cycle support Pregnancy support	Breast and ovarian cancers
VDR	Immune response regulation Bone health maintenance	Autoimmune diseases, cancer, and cardiovascular disorders
AR	Muscle development Voice deepening	Androgen insensitivity syndrome, prostate cancer
THR	Development regulation Lipid metabolism	Thyroid hormone resistance, hypo- and hyperthyroidism
RAR	Skin regulation Vision regulation	Congenital malformations and skin diseases

**Table 2:** QSTR significance test results.

Source	Value	Standard Error	T	Pr > |t|	Lower Bound (95%)	Upper Bound (95%)
Bioconcentration log 10						
Intercept	5.986	0.735	8.150	<0.0001	4.534	7.438
Cl Atoms	0.519	0.078	6.684	<0.0001	0.366	0.673
Globularity SVD	0.760	0.244	3.116	0.002	0.278	1.243
Van der Waals Surface	0.016	0.003	4.605	<0.0001	0.009	0.022
Van der Waals Volume	−0.030	0.006	−4.816	<0.0001	−0.043	−0.018
Shape Index	−1.086	0.199	−5.459	<0.0001	−1.479	−0.693
Stretch Bend	0.102	0.017	5.923	<0.0001	0.068	0.136
Synthetic Accessibility	−0.221	0.099	−2.245	0.026	−0.416	−0.026
Fathead Minnow LC50 log 10						
Intercept	2.499	0.357	6.991	<0.0001	1.792	3.205
Molecular Complexity	1.318	0.564	2.337	0.021	0.204	2.433
Log P	0.543	0.023	23.362	<0.0001	0.497	0.589
Stretch Bend	0.192	0.017	11.301	<0.0001	0.159	0.226
*Daphnia magna* LC50 log 10						
Intercept	16.189	2.554	6.338	<0.0001	11.140	21.238
Molecular Weight	−0.040	0.012	−3.248	0.001	−0.065	−0.016
Cl Atoms	1.687	0.444	3.799	0.000	0.809	2.564
Globularity Volume	−6.764	2.080	−3.252	0.001	−10.875	−2.653
Molecular Complexity	2.416	0.958	2.522	0.013	0.523	4.310
Non-1,4 VDW	0.012	0.004	3.085	0.002	0.004	0.019
Total Energy	−0.012	0.004	−3.021	0.003	−0.020	−0.004

## Data Availability

Data are contained within the article or in the Supplementary Materials.

## Supplementary Materials

The following supporting information can be downloaded at: https://www.mdpi.com/article/10.3390/toxics12080597/s1, Table S1: PCDDF Molecular descriptors; Table S2: Molecular docking results; Table S3: Molecular dynamics results.
